# Essential Role of *Cg*Erg6p in Maintaining Oxidative Stress Tolerance and Iron Homeostasis in *Candida glabrata*

**DOI:** 10.3390/jof9050579

**Published:** 2023-05-17

**Authors:** Daniel Elias, Nora Tóth Hervay, Marek Bujdos, Yvetta Gbelska

**Affiliations:** 1 Department of Microbiology and Virology, Faculty of Natural Sciences, Comenius University in Bratislava, Ilkovicova 6, 842 15 Bratislava, Slovakia; elias25@uniba.sk (D.E.); nora.toth@uniba.sk (N.T.H.); 2 Faculty of Natural Sciences, Institute of Laboratory Research on Geomaterials, Comenius University in Bratislava, Ilkovicova 6, 842 15 Bratislava, Slovakia; marek.bujdos@uniba.sk

**Keywords:** *Candida glabrata*, oxidative stress, iron homeostasis, *ERG6*

## Abstract

The human pathogenic fungus *Candida glabrata* is the second leading cause of candidemia, a life-threatening invasive mycosis. Clinical outcomes are complicated by reduced susceptibility of *C. glabrata* to azoles together with its ability to evolve stable resistance to both azoles and echinocandins following drug exposure. Compared to other *Candida* spp., *C. glabrata* displays robust oxidative stress resistance. In this study, we investigated the impact of *CgERG6* gene deletion on the oxidative stress response in *C. glabrata. CgERG6* gene encodes sterol-24-C-methyltransferase, which is involved in the final steps of ergosterol biosynthesis. Our previous results showed that the *Cgerg6Δ* mutant has a lower ergosterol content in its membranes. Here, we show that the *Cgerg6Δ* mutant displays increased susceptibility to oxidative stress inducing agents, such as menadione, hydrogen peroxide and diamide, accompanied with increased intracellular ROS production. The *Cgerg6Δ* mutant is not able to tolerate higher concentrations of iron in the growth media. We observed increased expression of transcription factors, *Cg*Yap1p, *Cg*Msn4p and *Cg*Yap5p, together with increased expression of catalase encoding the *CgCTA1* gene and vacuolar iron transporter *CgCCC1* in the *Cgerg6Δ* mutant cells. However, it seems that the *CgERG6* gene deletion does not influence the function of mitochondria.

## 1. Introduction

*Candida glabrata* is a haploid yeast belonging to the clade *Nakaseomyces*. Although it is found as a commensal in healthy individuals, *C. glabrata* is considered the second most common cause of candidiasis which is difficult to treat, resulting in high mortality in immune compromised patients [1,2]. Further complication of *C. glabrata* infections is its inherent increased tolerance to commonly used antifungal drugs. Owing to its reduced susceptibility to azole antifungals and emerging resistance to echinocandins, effective treatment of *C. glabrata* infections remains a clinical challenge.

In order to survive and successfully proliferate in different host niches that are complex and dynamic, *C*. *glabrata* must rapidly adapt to a diverse range of environmental stresses. The *C. glabrata* genome shows plasticity, and alterations in karyotype and chromosome size have been observed in clinical isolates and laboratory strains [3]. Moreover, *C. glabrata* is able to evade the host immune system due to chromatin remodeling, the presence of yapsins, the ability to subvert macrophage cytokine production and the ability to resist reactive oxygen species (ROS) production [4,5,6].

*C. glabrata* is also intrinsically less susceptible to various environmental stresses [3,7,8,9]. Among several *C. glabrata* virulence factors, remarkable high oxidative stress resistance has been described [8,9]. Oxidative stress is induced by increased production of ROS, including hydrogen peroxide, superoxide radicals (O_2_**^.^**) and hydroxyl radicals (OH^−^). Increased ROS production is deleterious to cells, and leads to damage of proteins, lipids and DNA [10,11]. Excessive exposure to ROS also occurs during the oxidative burst response of host’s phagocytes, and is critical for fungal, bacterial and viral clearances. Yeast cells respond to ROS by altering the expression of genes encoding antioxidant defense mechanisms and genes encoding enzymes which repair and detoxify the resulting cellular damage. The fungal response to oxidative stress is complex and necessitates distinct, but also collaborative involvement of several regulators, such as Yap1p, Skn7p, and Msn2p/Msn4p as well as the species-specific involvement of HOG (high-osmolarity glycerol) pathway. The HOG pathway, a MAP kinase (MAPK) pathway, has been revealed to be crucial in responding to a wide range of stress conditions frequently encountered by fungi in their common habitats [9,11,12].

*C. glabrata* has a robust oxidative stress response (OSR) system. OSR of *C. glabrata* consists of ROS scavengers, and includes enzymes, such as catalase *Cg*Cta1p or superoxide dismutases *Cg*Sod1p, *Cg*Sod2p and the non-enzymatic glutathione and thioredoxin systems, to keep ROS and cystein oxidation at physiological levels [8,13,14]. Gene expression of these system is under the control of well-known *Saccharomyces cerevisiae* transcription factors homologs called *Cg*Yap1p, *Cg*Skn7p, *Cg*Msn2p and *Cg*Msn4p [13,15]. Recently, Dupont et al. [16] studied the reasons for the diversity of sterols in different eukaryotic kingdoms and demonstrated the antioxidant role of specific fungal sterol, ergosterol, which protects the membrane lipids from oxidative perturbations.

Ergosterol is essential for fungi and its biosynthetic pathway is one of the main targets of contemporary antifungal strategies [17,18,19,20,21]. Although the main function of sterols is connected to the structural and functional properties of the plasma membrane, recent reports show that dynamic changes in sterol composition allow yeast cells to adapt to metabolic remodeling and environmental stresses [22,23,24]. Expression of ergosterol biosynthesis genes is tightly modulated by specific transcription factors. As sterol biosynthesis requires heme, the expression of genes involved in ergosterol biosynthesis is finely tuned with iron-related genes. The late part of ergosterol biosynthetic pathway include enzymes that utilize iron as redox cofactor (Erg11p, Erg5p, Erg25p, Erg3p) [24]. In *C. glabrata* planktonic cells, the lack of iron enhances membrane fluidity and diffusion consistent with the impairment in ergosterol biosynthesis [25]. Iron is an essential element for nearly all living organisms due to its incorporation in key metalloenzymes, mostly through iron–sulfur clusters and hemes. Iron is involved in a large number of cellular processes, including DNA synthesis, translation, mitochondrial respiration or lipid biosynthesis [26]. The observation that *C. glabrata* mutants exhibiting low growth in iron starvation conditions are also defective in macrophage survival suggest that iron uptake is crucial for *C. glabrata* intraphagosomal persistence [25]. However, excess of iron is highly toxic to the cells because it produces ROS through Fenton or Haber–Weiss reactions and is also able to replace copper and zinc in metalloproteins, thus altering their enzymatic activity. Therefore, strict regulation of iron uptake and utilization is needed [27,28]. Yeasts’ response to low or high iron is controlled via Aft1p, Aft2p and Yap5p transcription factors, respectively [27,29]. *C. glabrata* iron regulon comprises parts of *S. cerevisiae* regulation network and elements of other pathogenic fungi. Therefore, *C. glabrata* has evolved an iron homeostasis network which seems to be unique within the pathogenic fungi [29]. Other studies reported a link between altered sterol metabolism and mitochondrial (Fe–S) cluster biosynthesis or mitochondrial DNA maintenance [30,31]. Functional mitochondria play a vital role in regulating yeast physiology including energy production, lipid and cell wall biosynthesis, iron homeostasis and also participate in virulence and antifungal drug tolerance [32].

An important virulence factor of *C. glabrata* is its capacity to adhere to both biotic and abiotic surfaces. The adherence is considered a prerequisite for colonization and pathogenesis of fungal infections. In *C. glabrata*, adherence is mediated largely by the GPI-containing cell wall proteins—adhesins—belonging to the EPA family [4,33]. Puri et al. [34] showed that iron positively affects adhesion. Moreover, Srivastava et al. [35] reported that *C. glabrata* cells grown in high-iron environment upregulate major adhesin-encoding *EPA1* gene, have a decreased histone deacetylase activity and they display increased adherence to epithelial cells. Recently, it has been also shown that *EPA2* is induced by exposure to hydrogen peroxide, independent of subtelomeric silencing. This induction requires three transcription factors: Yap1p, Skn7p, and Msn2p/Msn4p [36,37]. The adhesion to abiotic and biotic surfaces also partially depends on cell surface hydrophobicity (CSH) [38]. Suchodolski et al. [38] demonstrated that *C. albicans* strains with different CSH have altered lipid metabolism due to changes in ergosterol biosynthesis.

Our previous work has shown that the absence of sterol-24-C-methyltransferase, (converting zymosterol to fecosterol in the ergosterol biosynthetic pathway), encoded by the *CgERG6* gene, has profound effect on *C. glabrata* antifungal drug susceptibility and plasma membrane properties. *CgERG6* gene deletion in *C. glabrata* leads to reduced content of ergosterol in the *Cgerg6Δ* mutant. Its plasma membrane is hyperpolarized and its fluidity decreased in comparison with that of the parental strain. The absence of *Cg*Erg6p affected the cell wall integrity and calcineurin signaling in *C. glabrata* [39]. In this work, we study the influence of *CgERG6* gene deletion on the oxidative stress response in *C. glabrata.* Observed changes in the *C. glabrata Cgerg6Δ* deletion mutant plasma membrane properties, the necessity of *Cg*Erg6p to maintain the proper redox state in *C. glabrata* cells together with the fact Erg6p is not involved in the cholesterol biosynthetic pathway in humans, make *Cg*Erg6p an useful target for a new generation of antifungal drugs.

## 2. Materials and Methods

### 2.1. Yeast Strains and Media

The *C. glabrata* strains used in this study were *Cglig4Δ lig4::HIS3 trp1* [40] (in further text designated as wt) and its isogenic derivate *Cglig4Δ erg6Δ lig4::HIS3 erg6::TRP1* [39] (appointed here as *Cgerg6Δ*). The strain *C. glabrata Cglig4Δ lig4*::*HIS3 trp1* [40] was kindly provided by Patrick van Dijck (KU Leuven, Leuven, Belgium). The *Cglig4Δ* strain in which the *CgLIG4* gene has been deleted was generated to improve the homologous recombination efficiency in *C. glabrata.* The phenotypic analysis showed that the *lig4* mutant strain behaves exactly as the wild type for all conditions tested [40]. *C. glabrata* cells were grown either in glucose-rich (YPD, 1% yeast extract, 2% bactopeptone, 2% glucose), or synthetic minimal (YNB) medium containing 0.67% yeast nitrogen base without aminoacids (YNB, BD Difco^TM^, Franklin Lakes, NJ, USA) supplemented with 2% glucose and the auxotrophic requirements required. For testing the cell´s ability to grow on non-fermentable carbon sources, we used rich media containing instead of glucose 2% glycerol (YPG), 2% ethanol (YPE) and 2% lactate (YPL). For solid media, 2 g per 100 mL of agar were added.

### 2.2. Susceptibility Assays

The susceptibility of the parental strain to various cytotoxic compounds was compared with that of the *Cgerg6Δ* deletion mutant. Susceptibility was determined using spot assay [41]. To assess *C. glabrata* susceptibility to various oxidative stress inducing agents, we plated the cells on the solid growth media containing different concentrations of hydrogen peroxide, menadione and diamide. The overnight cultures grown in YPD were diluted to 1 × 10^7^ cells/mL and their 10-fold dilutions were prepared. Serial dilutions in 5 μL aliquots were spotted on the glucose-rich (YPD) medium or synthetic minimal (YNB) medium supplemented with tryptophan and various concentrations of the compounds. The concentrations were as follows: diamide: 2, 5 mM; menadione: 0.1, 0.2, 0.4 mM; hydrogen peroxide: 0.05, 1%; and FeCl_3_: 0.5, 1, 3, 5 mM. The plates were incubated at 30 °C for 2 days.

### 2.3. Measurement of Intracellular ROS Production

The production of reactive oxygen species (ROS) was measured using the probe dihydrofluorescein diacetate (H_2_DCFDA, Sigma-Aldrich, Saint Louis, MO, USA), which produces fluorescence being attacked by intracellular ROS. The cell-permeant H_2_DCFDA is a chemically reduced form of fluorescein used as an indicator for ROS in cells, for example, to detect the generation of reactive oxygen intermediates. Upon cleavage of the acetate groups via intracellular esterases and oxidation, the nonfluorescent H_2_DCFDA is converted to the highly fluorescent 2′,7′-dichlorofluorescein (DCF). We evaluated the intensity of cellular fluorescence as an estimate of the amount of free radicals produced in the cells, which was measured using the fluorometric method. The yeasts cells were grown to late exponential phase in glucose-rich (YPD) medium. Cells were washed in phosphate-buffered saline (PBS, pH 7). A suspension of 1 × 10^5^ cells in 100 μL samples were loaded in triplicate in a 96-well plate and incubated with 25 μM H_2_DCFDA. To induce ROS production in the cells, 0.5 μM menadione (Sigma-Aldrich, Saint Louis, MO, USA; dissolved in DMSO) was added. The DCF fluorescence signal was measured using GloMax Discover Microplate Reader (Promega Corp., Walldorf, Germany) at 0, 30, 60 and 90 min at excitation and emission wavelengths of 475 and 500–550 nm, respectively. For statistical analyses, unpaired Student *t*-test was used (significance level *p* ˂ 0.05).

### 2.4. Quantitative Real-Time PCR

The levels of gene transcripts were assessed using quantitative real-time PCR. Overnight cultures of yeast cells resuspended into glucose-rich (YPD) medium were grown to mid-logarithmic phase. Yeast cells were lysed using acid-washed glass beads and total RNA was extracted from cells using the hot acid phenol method. Purity and integrity of isolated total RNA was assessed using spectrophotometry and agarose gel electrophoresis, respectively. Further, total RNA samples were treated with DNase I, RNase-free (ThermoFisher Scientific, Frankfurt am Main, Germany) to remove contaminating genomic DNA according to the manufacturer’s instructions. First-strand cDNA was synthetized from 1 μg of total RNA using 200 U of the Revert AidTMH Minus M-MuLV Reverse transcriptase (ThermoFisher Scientific, Frankfurt am Main, Germany). Quantitative real-time PCRs were performed in triplicate using the Applied Biosystems 7900 HT Fast Real-Time PCR system. Independent PCRs were performed using the same cDNA for both the gene of interest and the *CgACT1* gene, via the HOT FIREPol^®^ EvaGreen^®^ qPCR Mix Plus (ROX) (Solis BioDyne, Tartu, Estonia) and used to quantify the expression of genes involved in oxidative stress response (*CgYAP1*, *CgSKN7*, *CgMSN4*, *CgCTA1*, *CgSOD1*, and *CgSOD2*) or iron metabolism (*CgAFT1*, *CgAFT2*, *CgYAP5*, *CgFTR1*, *CgCCC1*, and *CgISU1*). The qRT-PCR were performed in triplicates. The *CgACT1* mRNA level was used as the internal control. The relative value obtained for each target gene in the parental strain was set as 1 and the remaining values are relative to that value. Primers used to perform qRT-PCR experiments are listed in Table S1.

## 3. Results

### 3.1. CgErg6p Is Involved in Candida glabrata Oxidative Stress Response

**Figure 1:**
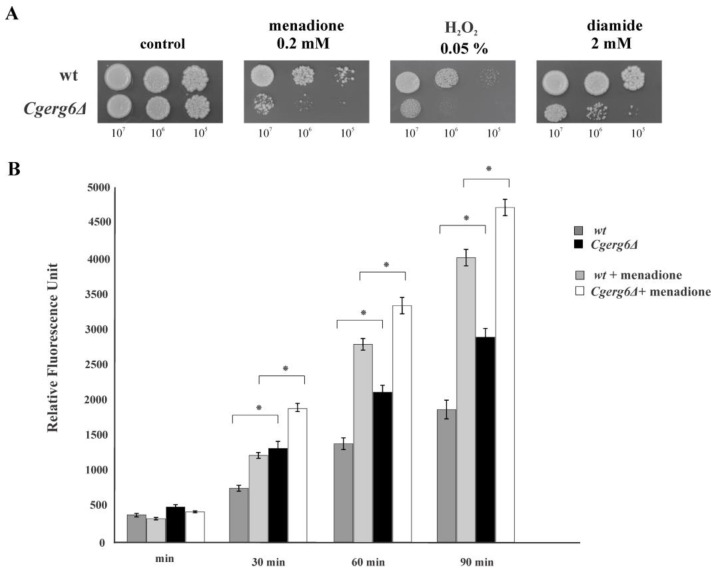
(**A**) Growth of parental strain (wt) and *Cgerg6Δ* mutant in the presence of representative concentration of oxidants. Tenfold serial dilutions of overnight cultures were prepared, 5 µL aliquots spotted onto YPD plates and incubated at 30 °C for 2 days; (**B**) Production of ROS by *C. glabrata* parental strain and *Cgerg6Δ* mutant in the presence or absence of 0.5 μM menadione. A suspension of 1 × 10^5^ cells in 100 μL samples was analyzed, and incubated with 25 μM H_2_DCFDA. The DCF fluorescence signal was measured using GloMax Discover Microplate Reader at 0, 30, 60, 90 min at excitation and emission wavelengths of 475 and 500–550 nm, respectively * statistical significance (*p* ˂ 0.05).

To ensure successful infection, *C. glabrata* has evolved several virulence factors, among them, a robust oxidative stress response. The ability of *Cgerg6Δ* deletion mutant to grow in the presence of hydrogen peroxide, menadione and diamide is markedly affected compared with the growth of isogenic parental strain (Figure 1A).

Oxidative stress is generated by ROS, the endogenous by-products of aerobic metabolism arising from mitochondria and the oxidative metabolism in peroxisome and the endoplasmic reticulum. Therefore, in the next experiment we evaluated the endogenous ROS production in the *Cgerg6Δ* deletion mutant and its parental wild-type strain. As Figure 1B shows, the amount of endogenous ROS is significantly higher in the *Cgerg6Δ* deletion mutant as compared to the parental strain. The incubation of both strains in the presence of menadione leads to further increase in ROS production and the effect of menadione is more pronounced in the *Cgerg6Δ* deletion mutant (Figure 1B).

**Figure 2:**
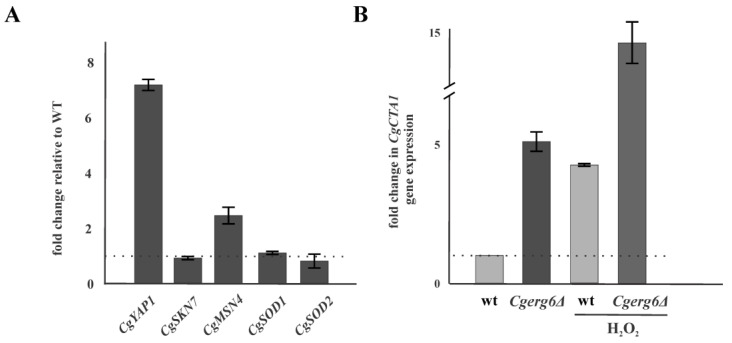
Relative gene expression levels in *C. glabrata Cgerg6Δ* deletion mutant compared with those in parental strain (wt). (**A**) The mRNA expression levels in *C. glabrata Cgerg6Δ* deletion mutant for the transcription factors *CgYAP1*, *CgSKN7*, *CgMSN4* and genes encoding superoxide dismutases *CgSOD1*, and *CgSOD2* (**B**) Relative gene expression levels for the catalase encoding gene *CgCTA1* in *C. glabrata* wt and *Cgerg6Δ* deletion mutant. The gene transcript levels in non-treated parental strain were set as 1. For induction, the cells were incubated with 0.01% hydrogen peroxide for 1 h. The results are expressed as mean values of three independent experiments (±standard deviation) normalized to the β-actin mRNA level.

Phagocytic cells generate ROS to eliminate internalized pathogens. However, pathogens have coopted several enzymatic and non-enzymatic mechanisms to eliminate ROS. We used quantitative reverse transcription-PCR (qRT-PCR) to analyze the mRNA expression levels for the transcription factors *CgYAP1*, *CgSKN7* and *CgMSN4* involved in *C. glabrata* oxidative stress response as well as the mRNA expression levels for *CgCTA1*, *CgSOD1* and *CgSOD2* genes encoding ROS detoxifying enzymes. Figure 2A shows that the mRNA level of *CgYAP1* and *CgMSN4* genes, encoding the general stress response transcription factor, is significantly induced in the *Cgerg6Δ* mutant compared with that of the parental strain. The absence of *Cg*Erg6p did not markedly influence the expression level of the yeast transcription factor involved in oxidative stress response, *CgSKN7* (Figure 2A). The mRNA level of *CgCTA1*, the single gene encoding catalase in the *C. glabrata* genome, is four times higher in the Cgerg6Δ deletion mutant compared with that in the parental strain. Incubation of cells in the presence of hydrogen peroxide for 1 h before the mRNA isolation led to further increase in the *CgCTA1* gene expression in both the strains, the *Cgerg6Δ* deletion mutant and the parental strain (Figure 2B). The transcript levels of genes encoding superoxide dismutases *CgSOD1* and *CgSOD2* are not changed in the strains studied (Figure 2A).

### 3.2. CgErg6p Is Not Necessary for Mitochondrial Function in C. glabrata

**Figure 3:**
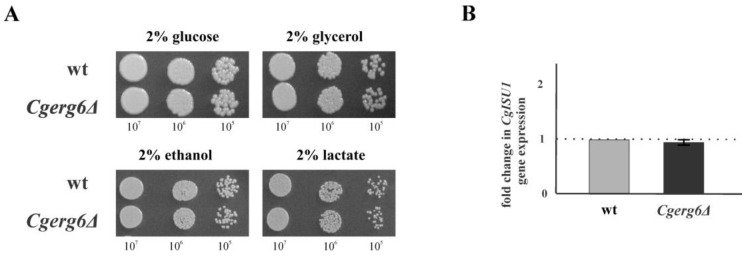
(**A**) Growth of parental strain (wt) and *Cgerg6Δ* mutant in the presence of glucose and non-fermentable carbon sources. Tenfold serial dilutions of overnight cultures were prepared, 5 µL aliquots spotted onto YPD, YPG, YPE and YPL plates and incubated at 30 °C for 2 days; (**B**) The mRNA expression levels for the *CgISU1* gene, encoding an iron-binding protein of the mitochondrial matrix involved in the Fe–S cluster assembly. The gene transcript levels in parental strain were set as 1. The results are expressed as mean values of three independent experiments (±standard deviation) normalized to the β-actin mRNA level.

Mitochondria are considered to be the main source of endogenous ROS production in aerobic cells. Recent reports demonstrated a link between impaired ergosterol biosynthesis and the loss of mitochondrial DNA in *S. cerevisiae* [31]. Therefore, in the next experiment we analyzed the ability of our strains to grow in the presence of nonfermentable carbon sources. As Figure 3A shows, the *CgERG6* gene deletion does not abolish the growth of *Cgerg6Δ* deletion mutant on solid media containing glycerol, ethanol or lactate. Furthermore, qRT-PCR analysis did not reveal any changes in the expression of *CgISU1* gene, which encodes conserved protein of the mitochondrial matrix by performing a scaffolding function during the assembly of Fe–S clusters, in the *Cgerg6Δ* deletion mutant (Figure 3B).

### 3.3. CgERG6 Gene Deletion Impairs Iron Homeostasis in the Cgerg6Δ Mutant

**Figure 4:**
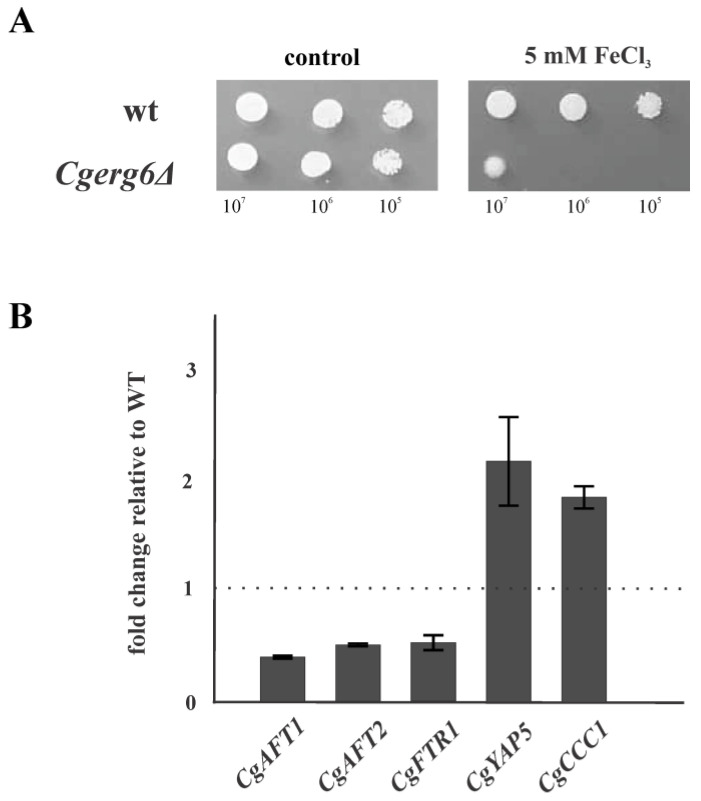
(**A**) Growth of parental strain (wt) and *Cgerg6Δ* mutant in the presence of 5mM FeCl_3_. Tenfold serial dilutions of overnight cultures were prepared, 5 µL aliquots spotted onto YNB plates and incubated at 30 °C for 2 days; (**B**) The mRNA expression levels for the genes involved in iron homeostasis. The gene transcript levels in parental strain were set as 1. The results are expressed as mean values of three independent experiments (±standard deviation) normalized to the β-actin mRNA level.

To maintain iron homeostasis, yeast cells must get enough iron for essential cellular processes and resist toxic iron excess. The redox-active properties of iron can be deleterious to cells if excess iron is present, as it participates in the Fenton reaction, which generate hydroxyl radicals and other ROS that can damage most cellular macromolecules. In the next experiment, we explored the ability of *Cgerg6Δ* deletion mutant to grow in the presence of elevated concentrations of extracellular iron. As Figure 4A shows, the growth of *Cgerg6Δ* deletion mutant in the presence of FeCl_3_ is very poor compared with that of the wild-type strain. We did not observe any difference in growth of the strains on the plates containing 0.5 mM and 1 mM FeCl_3_. The most prominent difference was seen on the plate containing 5 mM FeCl_3_.

To explore the reason of the poor growth of the *Cgerg6Δ* deletion mutant in the presence of FeCl_3_, we examined the mRNA levels of *CgAFT1* and *CgAFT2* genes, encoding transcription factors involved in iron utilization and homeostasis, and the *CgFTR1* gene, encoding the high affinity iron permease. The loss of *CgERG6* gene leads to twofold decrease in the mRNA level of *CgAFT1*, *CgAFT2* and *CgFTR1* genes compared to that in the parental wild-type strain (Figure 4B). Moreover, the transcript levels of *CgYAP5* (bZIP iron sensing transcription factor) and *CgCCC1* (vacuolar Fe^2+^ transporter) genes increased roughly twofold in the *Cgerg6Δ* deletion mutant (Figure 4B).

## 4. Discussion

Sterols are essential lipids of eukaryotic cells with important structural and signaling functions. Sterols contribute to fluidity, permeability, microdomain formation and membrane activities in yeast cells [42,43]. It was proposed that the evolution of sterol synthesis pathways could be linked to the oxygenation of Earth [44], highlighting the possible role of sterol evolution in the management of oxidative stress [45,46]. In this work, we demonstrate that the *CgERG6* gene, encoding sterol-24-C-methyltransferase, is required for oxidative stress tolerance in the opportunistic yeast pathogen, *C. glabrata*. The results obtained show increased susceptibility of *Cgerg6Δ* deletion strain to oxidative stress inducing agents. The absence of transmethylation reaction in the ergosterol biosynthetic pathway compromised the growth of *C. glabrata* mutant cells in the presence of hydrogen peroxide, menadione and diamide. *Cg*Erg6p is therefore essential for optimal oxidative stress adaptation in *C. glabrata*. The loss of *CgERG6* gene could lead to increased endogenous ROS production or compromises the ability of *C. glabrata* to detoxify ROS. Measurement of endogenous ROS production showed increased ROS production in the *Cgerg6Δ* deletion mutant compared with that in the wild-type strain. The observed susceptibility of *Cgerg6Δ* deletion mutant to oxidative stress inducing agents could thus be the result of increased endogenous ROS production. However, we cannot exclude the possibility that changes in the plasma membrane permeability, induced by *CgERG6* gene deletion, could lead to inability of mutant cells to adapt to oxidative stress. This proposition corresponds with the observation of Folmer et al. [47], who have shown that changes in the plasma membrane biophysical properties influence the adaptability of *S. cerevisiae* to oxidative stress. Our previous report demonstrated substantial changes in plasma membrane permeability as a result of *CgERG6* gene deletion [39].

The observed changes in the susceptibility to oxidative stress inducing agents in our *Cgerg6Δ* deletion mutant may also reflect diminished capacity of mutant cells to detoxify ROS. In oxidative stress conditions, changes in expression of genes encoding ROS detoxifying enzymes were reported [8]. Several reports showed that *C. glabrata* contains a single *CgCTA1* gene encoding catalase, belonging to the core response to oxidative stress in this pathogen [8,9,13]. The expression of *CgCTA1* gene is induced in the absence of *CgERG6* gene as well as in the wild-type strain incubated in the presence of hydrogen peroxide before the mRNA isolation. The *CgERG6* gene deletion did not lead to observable changes in *CgSOD1* and *CgSOD2* genes expression. *C. glabrata, CgSOD1* and *CgSOD2* in contrast to *S. cerevisiae*, *Schizosaccharomyces pombe* and *Candida albicans*, are constitutively expressed even in the presence of oxidative stress and their expression is independent of the *Cg*Yap1p transcription factor [8,9].

Expression of ROS detoxifying enzymes is controlled by several transcription factors: *Cg*Yap1p, *Cg*Skn7p and the general stress transcription factors *Cg*Msn2p/*Cg*Msn4p [13,48]. The Msn2p/Msn4p bind to the CCAAT consensus sequence in the promoters of their target genes. The observed increased expression of *CgYAP1* and *CgMSN4* genes in the *Cgerg6Δ* deletion mutant might point to the fact that the loss of *CgERG6* gene induces a more global stress effect in the *C. glabrata* cells. In our previous work, we showed reduced thermotolerance and reduced tolerance to osmotic stress of the *Cgerg6Δ* deletion mutant [39]. The stress-regulatory transcription factors, Msn2p/Msn4p, influence the expression of more than 90% of the genes that are up-regulated during heat stress, osmotic stress, and carbon starvation stress [49] and they regulate fatty acid oxidation in budding yeast [50].

Ergosterol production has been shown to be essential for mitochondrial DNA maintenance in *S. cerevisiae* [31]. Another study has shown that the deletion of *ERG29* gene in *S. cerevisiae* results in decreased ability of mitochondria to synthetize Fe–S clusters [30]. Ergosterol, as an essential structural component of membranes, influences the function of enzymes localized in inner and outer mitochondrial membrane including respiration. *Erg3Δ* deletion mutant in *S. cerevisiae* is unable to grow on the respiratory substrates [51,52]. However, the mitochondria of our *Cgerg6Δ* deletion mutant seem to be functional: the mutant is able to grow on nonfermentable carbon sources and the expression of *CgISU1* gene involved in Fe–S cluster biosynthesis in mitochondria does not change compared to that in the isogenic parental strain.

Several studies also reported that genes involved in ergosterol biosynthesis are regulated by iron availability [23,24,53]. The late biosynthetic pathway enzymes utilize iron as redox cofactor (the hemoproteins Erg11p, and Erg5p; and the oxo-diiron enzymes Erg25p, and Erg3p). We observed that the growth of *Cgerg6Δ* deletion mutant is compromised in the presence of increased extracellular iron content. *C. glabrata* has conserved orthologues for most of the *S. cerevisiae* iron homeostasis genes. One striking difference between *C. glabrata* and other yeast species is the relatively low number of ferric reductases encoded in its genome [27]. This fact could contribute to the compromised growth of mutant cells in the presence of ferric ion. *C. glabrata* has also been reported to respond to iron-replete conditions by trafficking the high-affinity iron permease *Cg*Ftr1p from the plasma membrane to the vacuole. This retrograde trafficking is dependent on the sole class III phophoinositide-3-kinase kinase, *Cg*Vps34p [54]. Despite the decreased expression of *CgAFT1*, *CgAFT2* and *CgFTR3* genes belonging to iron acquisition regulon, no statistically significant changes in intracellular iron content in the *Cgerg6Δ* deletion mutant compared to the parental strain (Table S2) was observed. Iron-sensitive strains are less thermotolerant [39] and may shut down iron import, but they are unable to detoxify iron properly, possibly due to defects in the sequestration machineries [55]. Excess of intracellular iron promotes the generation of ROS via hydroxyl radical production through Fenton and Haber–Weiss reactions [28,56]. In fact, the cytosol in iron-sensitive *Saccharomyces* yeast strains was found to be generally more oxidized than in resistant strains grown at the same iron concentrations [55]. To cope with iron overload, yeast cells decrease iron uptake and increase iron storage in both vacuole and proteins. Three bZip transcription factors, *Cg*Yap1p, *Cg*Yap5p and *Cg*Yap7p, have also been implicated in regulation of the heme biosynthesis, iron-excess stress response and iron–sulfur cluster biosynthesis, respectively [27]. However, a very important part of the transcriptional response to iron excess is handled by the iron-responsive regulator, Yap5p. Yap5p is a transcription factor of the bZip family which recognizes the Yap Response Element (YRE) TTA(C/G)TAA in the promoters of its target genes. Yap5p plays an important role in the overexpression of *CCC1* gene, which is involved in the main route of iron detoxification [57]. At high iron levels, the yeast Yap5p together with Msn2p/Msn4p transcription factors activate the expression of a vacuolar iron importer Ccc1p, which is the most important high-iron protecting factor devoted to detoxify excess of cytosolic iron that is stored in the vacuole [57,58,59]. In *C. glabrata,* CHIP-seq and transcriptome analyses have shown that CCAAT binding complex actually plays a dual role; as a constitutive activator of the respiratory genes and as an activator of the *Cg*Yap5p regulon upon iron overload [60]. In *C. glabrata,* both the CCAAT and the YRE motifs are necessary for proper regulation of *Cg*Yap5p targets [27]. We have shown increased expression of both *CgYAP5* and *CgMSN4* genes encoding transcription factors as well as their target, the *CgCCC1* gene, thus corroborating the result of higher intracellular iron content in the *Cgerg6Δ* deletion mutant grown in the presence of high concentration of iron (Table S2).

The increased incidence of invasive mycoses and the problem of antimicrobial resistance together with the limited efficacy of current antifungal agents have motivated the search for new drugs. Development of invasive *Candida* infection depends on the capacity of the fungal pathogen to evade host immune system. During the infection, phagocytic cells (neutrophils and macrophages) engulf yeast cells and initiate the production of ROS. This process is called an oxidative burst. In fact, phagocytic cells represent the first line of defense in fungal infections [7,8]. As the *ERG6* deletion leads to changes in redox state, the compound directly affecting sterol C24 methyltransferase could be effective in combating invasive candidiasis.

## 5. Conclusions

Our results demonstrate that the *CgERG6* gene is necessary for oxidative stress tolerance and iron homeostasis in *C. glabrata*. Overall, our obtained results suggest that *Cg*Erg6p is required to maintain the proper redox state in *C. glabrata* cells. Our data also reveal new role of *Cg*Erg6p and provide more knowledge on the identification of new potential antifungal targets.

## Data Availability

Not applicable.

## Supplementary Materials

The following supporting information can be downloaded at: https://www.mdpi.com/article/10.3390/jof9050579/s1, Table S1: List of oligonucleotides; Table S2: Intracellular iron content in *C. glabrata* strains.
