# Supplementary material for: Essential Role of CgErg6p in Maintaining Oxidative Stress Tolerance and Iron Homeostasis in Candida glabrata
Source: J Fungi (Basel). 2023 May 17;9(5):579. doi: 10.3390/jof9050579 (PMC10219032; doi:10.3390/jof9050579)
Supplement: Supplementary file 1 [file jof-09-00579-s001.zip › jof-2389388-supplementary.pdf]

Supplementery material

Table S1. List of oligonucleotides

CgACT1 F	TTCAACGTTCCAGCCTTCT
CgACT1 R	GTAACACCGTCACCAGAGT
CgYAP1 F	GTGGACTTGGAGAACTTGAC
CgYAP1 R	ACTTCCGCAACTCTTTCAC
CgMSN4 F	CATTAATCCCGTATTGCTGTCC
CgMSN4 R	CTTCGTTGTCGTAATCATTGCC
CgCTA1 F	ACAAACTAACTTGAAGGACCCA
CgCTA1 R	GACATAGTGCCATTCACCCT
CgSOD1 F	CCACTATCACCTACGAGATTGC
CgSOD1 R	CACCGACGTGTCTGTTCTC
CgSOD2 F	CACTACTCCAAGCACCACC
CgSOD2 R	TTCGTGTACCCACCTCCA
CgSKN7 F	ATGAGCCCTTCATTGCCT
CgSKN7 R	TGGCGATTCAGTTTGTCCT
CgAFT1 F	TACCCGCAGGGAATAGAGATCG
CgAFT1 R	TTCATGTTCCCTAGCCAAAGCC
CgAFT2 F	GCCATGAACCAAAGGACCTACG
CgAFT2 R	GTAGAGAAAGTACACCGCACCC
CgYAP5 F	GTTGATCTGGATGGTCTGAAGG
CgYAP5 R	TCGGAGTAATTGAAGGTGAGC
CgCCC1 F	GTTCAGCAATGTGGACCCGC
CgCCC1 R	GATCACCAGCTTCGCGTCAC
CgFTR1 F	AGCCGGTTTGTTCTCAAGAGG
CgFTR1 R	CACCACCTTCAGAAGCATCACC
CgISU1 F	CGTCCGTTTACCAGGATGTACC
CgISU1 R	GTTCCGCAGAGTCTTGTCCA

Table S2. Intracellular iron content in *C. glabrata* strains

The amount of Fe in the samples was measured using an atomic absorption spectrometer in flame emission mode (Perkin Elmer 1100). Cells were incubated in the absence or presence of 1mM $FeCl_3$. * P <0,001

	**µg Fe / 10^{10} cells**	
**$FeCl_3$**	**wt**	***Cgerg6Δ***
**-**	**7.3 ± 0.0**	**6.1 ± 0.1**
+	**576.5 ± 2.5***	**1305 ± 25***
